# Oligodendrocytes in amyotrophic lateral sclerosis and frontotemporal dementia: the new players on stage

**DOI:** 10.3389/fnmol.2024.1375330

**Published:** 2024-03-22

**Authors:** Marguerite Jamet, Luc Dupuis, Jose-Luis Gonzalez De Aguilar

**Affiliations:** Strasbourg Translational Neuroscience and Psychiatry, Institut National de la Santé et de la Recherche Médicale, Université de Strasbourg, Strasbourg, France

**Keywords:** amyotrophic lateral sclerosis, frontotemporal dementia, myelin, neurodegenerative disease, oligodendrocyte

## Abstract

Amyotrophic lateral sclerosis (ALS) and frontotemporal dementia (FTD) are fatal adult-onset neurodegenerative disorders that share clinical, neuropathological and genetic features, which forms part of a multi-system disease spectrum. The pathological process leading to ALS and FTD is the result of the combination of multiple mechanisms that operate within specific populations of neurons and glial cells. The implication of oligodendrocytes has been the subject of a number of studies conducted on patients and related animal models. In this review we summarize our current knowledge on the alterations specific to myelin and the oligodendrocyte lineage occurring in ALS and FTD. We also consider different ways by which specific oligodendroglial alterations influence neurodegeneration and highlight the important role of oligodendrocytes in these two intrinsically associated neurodegenerative diseases.

## 1 A brief overview on amyotrophic lateral sclerosis and frontotemporal dementia

Amyotrophic lateral sclerosis (ALS) and frontotemporal dementia (FTD) are fatal neurodegenerative disorders. ALS is the most frequent motor neuron disease in adults and is primarily characterized by the degeneration of upper and lower motor neurons, progressive muscle wasting, fasciculations, dysarthria, dysphagia, altered reflexes and spasticity, leading to total paralysis and death within two to five years after diagnosis (Hardiman et al., 2017; Feldman et al., 2022). Most cases are sporadic, with a family history being observed in 10% of patients. The most common genetic causes identified until now account for about 70% of familial ALS and include expansions of a hexanucleotide repeat in chromosome 9 open reading frame 72 (*C9orf72*), involved in endosomal trafficking and autophagy; mutations in superoxide dismutase 1 (*SOD1*), encoding an antioxidant enzyme; and transactive response DNA binding protein 43 (*TARDBP*) and fused in sarcoma (*FUS*), which encode the DNA/RNA binding proteins TDP43 and FUS, respectively. Additional genes have been associated with ALS although they only account for a minority of cases (Ghasemi and Brown, 2018).

Frontotemporal dementia (FTD) is the second most common cause of presenile dementia and is primarily characterized by the degeneration of the frontal and temporal lobes, leading to two main clinical manifestations: progressive alterations in social behavior and personality, known as behavioral variant FTD; and language and speech difficulties, referred to as primary progressive aphasia. Life expectancy ranges from 7 to 13 years from onset in cases that do not have motor involvement (Grossman et al., 2023). A family history is observed in 40% of patients. Known major genetic causes include *C9orf72* repeat expansions, and mutations in progranulin, involved in lysosomal function and protein trafficking, and microtubule-associated protein Tau (*MAPT*), responsible for microtubule assembly and stability. Other causative or susceptibility genes have also been identified (Antonioni et al., 2023). Taken together, these reports emphasize the importance of the genetic overlap between ALS and FTD, with *C9orf72* repeat expansions being the most common genetic cause responsible for about 10% of all ALS and FTD cases (Abramzon et al., 2020; Tang et al., 2020). Besides common genetics, ALS and FTD also share clinical and neuropathological features. Up to 50% of ALS patients exhibit alterations in behavior and cognition reminiscent or typical of FTD, and around 30% of FTD patients meet criteria of ALS-like motor impairment (van Es et al., 2017). At the histological level, TDP43, irrespective of the presence or absence of *TARDBP* mutations, forms aggregates delocalized in the cytoplasm of affected populations of neurons and glial cells in the vast majority of ALS cases and in almost the half of FTD cases (Neumann et al., 2006). It is therefore now commonly accepted that ALS and FTD are the two ends of a multi-system disease spectrum.

## 2 Some notes on the physiology of oligodendrocytes

Based on the genetic diversity of ALS and FTD, multiple pathogenic mechanisms have been proposed to contribute to neurodegeneration, although the precise nature of the selective loss of specific populations of neurons remains elusive. Furthermore, since not only neurons but also other cells in the central nervous system (CNS) are affected by the pathological process, the situation is even more complex than previously thought, adding considerable complexity to its understanding (Ling et al., 2013; Hardiman et al., 2017; Mejzini et al., 2019; Abramzon et al., 2020; Antonioni et al., 2023). Growing evidence has accumulated during the last decade supporting the implication of oligodendrocytes, which are the myelinating cells in the CNS responsible for trophic support and the rapid propagation of electrical signals along the axons. The development of oligodendrocytes starts at the end of the embryonic period, when committed oligodendrocyte progenitor cells (OPCs) from the ventricular zones in the brain and spinal cord intensively proliferate and migrate to invade the entire CNS according to precisely regulated mechanisms. A few weeks after birth, OPCs begin to differentiate into mature oligodendrocytes (Kuhn et al., 2019). The interplay between axon needs and mature oligodendrocytes depends on the amount and localization of OPCs (Emery, 2010). The extent of OPC proliferation and migration also influences the density and repartition of interneurons during late embryonic stages (Fang and Bai, 2023). Therefore, impairments in OPC development may bring about important consequences for mature oligodendrocyte localization and availability as well as for neuronal excitatory/inhibitory balance.

Once they are differentiated, the majority of oligodendrocytes insulate axons with a multilayered sheath wrapped around them. This structure consists of internodes of individual myelin segments separated by tiny unmyelinated gaps called nodes of Ranvier. One oligodendrocyte is able to myelinate segments of up to 50 different axons. The myelin sheath contains around 70% lipids (of dry mass), of which major lipid components include 40% cholesterol, 40% glycerophospholipids and 20% glycosphingolipids. The amount of cholesterol is twice as important as in other cellular membranes, and its metabolic regulation is instrumental in forming and maintaining the myelin sheath. Proteins represent 30% of the dry myelin mass. Proteolipid protein (PLP) is the most abundant protein of CNS myelin and plays a central role in the formation of its multilamellar structure. Myelin basic protein (MBP) is the second most abundant protein and is essential for the compaction of the myelin sheath. Other important proteins include 2′,3′-cyclic-nucleotide 3′-phosphodiesterase (CNP), which contributes to the formation of cytoplasmic open spaces within the myelin sheath to allow for local translation of *MBP* mRNA; myelin-associated glycoprotein (MAG) and myelin oligodendrocyte glycoprotein (MOG), two adhesion molecules mostly involved in cell-cell interactions; and myelin-associated oligodendrocyte basic protein (MOBP) which, similarly to MBP, seems to participate in myelin compaction (Han et al., 2013; Stadelmann et al., 2019).

The presence of a compacted myelin sheath around the axon prevents current leakage and allows electrical impulses to transmit faster thanks to a phenomenon called saltatory conduction, whereby the current flows longitudinally along the myelinated segments of the axon, generating axon potentials that “jump” from one node of Ranvier to the next one. This process of saltatory conduction may be subject to changes, since the myelin sheath is a dynamic structure able to receive signals from the axon and adapt its characteristics to axonal needs (Almeida and Lyons, 2017). By modifying the length of the internodes or thickness of the myelin sheath, oligodendrocytes control the speed of conduction along the axons and, subsequently, the synchrony of signals between distant neuronal locations. This synchrony between multiple synaptic inputs is essential for proper function, as if inputs are not received coincidentally they will not be integrated by the post-synaptic neuron, possibly leading to dysfunctional neuronal circuits (Kato et al., 2020). Besides their role in the propagation of electrical impulses, oligodendrocytes are also meant to metabolically support the axons by providing monocarboxylate substrates, such as lactate, through the specific oligodendroglial monocarboxylate transporter MCT1, which helps maintain axonal activity and ensure neuronal survival (Nave et al., 2023).

Pathological alterations in oligodendrocytes and/or myelin have a direct impact on neuronal activity, as shown in the context of several neurodegenerative conditions, such as multiple sclerosis, multiple system atrophy, Alzheimer’s disease and Parkinson’s disease (Han et al., 2022). The purpose of this review is to summarize our current knowledge of the alterations specific to myelin and the oligodendrocyte lineage occurring in ALS and FTD. However, we do not address those alterations affecting several cell types simultaneously, such as, for instance, defective autophagy or mitochondrial dysfunction. We recommend readers to refer to recent articles published elsewhere that address these topics in detail (Anoar et al., 2021; Chua et al., 2022; Motataianu et al., 2022). Finally, we consider the different ways that specific oligodendroglial alterations influence neurodegeneration in ALS and FTD, highlighting the important role of oligodendrocytes in these two intrinsically associated neurodegenerative diseases.

## 3 Pathological hallmarks of mature oligodendrocytes in ALS and FTD

### 3.1 Protein aggregation in mature oligodendrocytes

Neuronal cytoplasmic inclusions have been extensively described in ALS and FTD, but many studies have also reported the presence of inclusions in oligodendrocytes, as well as other glial cells. In general, these oligodendroglial inclusions mainly appear in the ventral horns of the spinal cord, corticospinal tracts and frontal cortex, which are the most affected areas in ALS or FTD, and they are essentially found around the nucleus and periaxonal cytoplasm (Stieber et al., 2000; Fatima et al., 2015; Ferraiuolo et al., 2016; Tables 1, 2).

**TABLE 1:** Alterations observed in ALS and FTD patients.

Patient	Mutation	Inclusions in mature oligodendrocytes	OPCs	Myelin	References
ALS	Sporadic and familial cases	TDP43 and misfolded SOD1	Reactive changes and aberrant proliferation	Water accumulation; diffuse myelin pallor and demyelination; decrease in MBP *, sphingomyelin and *HMGCR* mRNA; and increase in cholesterol esters	Hayashi et al., 2001; Cutler et al., 2002; Kang et al., 2013; Kolind et al., 2013; Philips et al., 2013; Brettschneider et al., 2014; Rohan et al., 2014; Fatima et al., 2015; Blasco et al., 2017; Ho et al., 2021; Sadler et al., 2022
ALS	*SOD1*			Decrease in MBP in motor cortex and spinal cord gray matter	Kang et al., 2013
ALS	Optineurin			Myelin pallor and spongiosis in motor cortex	Nolan et al., 2021
ALS	Angiogenin	TDP43, ubiquitin and p62			Seilhean et al., 2009
ALS/FTD	Sporadic and familial cases			Decrease in *MOBP* mRNA	Humphrey et al., 2023
ALS/FTD	*C9orf72*	p62, TDP43 and expanded RNA repeats into cytoplasmic RNA foci	Increase in number	Demyelination in motor cortex and lumbar spinal cord; and decrease in MBP and sphingolipid in cortex but no alteration in cholesterol	DeJesus-Hernandez et al., 2011; Kang et al., 2013; Mizielinska et al., 2013; Kovacs et al., 2016; Lorente Pons et al., 2020; Marian et al., 2023
FTD	Sporadic and familial cases	Tau protein, TDP43, p62, LDLR, FUS	Increased oligodendroglial signature	Decrease in gene expression related to cholesterol metabolism	Neumann et al., 2009; Kahlson and Colodner, 2016; Hosokawa et al., 2017; Ho et al., 2021; Hasan et al., 2022
FTD	Sequestosome 1	TDP43, p62			Kovacs et al., 2016
FTD	Progranulin			Decrease in MBP, CNP, PLP and sphingolipids; and increase in cholesterol esters in white matter	Marian et al., 2023
FTD	*TMEM106B*			Decrease in sphingolipids	Lee et al., 2023
IPSC-derived oligodendrocytes of ALS patients	*TARDBP*	TDP43			Barton et al., 2021
IPSC-derived oligodendrocytes of ALS patients	*SOD1*, *FIG4*, *C9orf72*	Misfolded SOD1			Ferraiuolo et al., 2016

*MBP content was normal in white matter, according to Sadler et al. (2022).

**TABLE 2:** Alterations observed in animal models of ALS and FTD.

Animal model	Feature	Mature oligodendrocytes	OPCs	Myelin	References
*SOD1^G93A^* mice	Mice carrying human G93A *SOD1* mutation	TDP43, SOD1, MBP and PLP inclusions; thicker cell bodies; elongated and hypertrophic morphology; expression of apoptotic marker; and reduced number	Aberrant proliferation and impaired differentiation	Decreased g-ratio, water accumulation, vacuolization, lamellae detachment, loss of compaction and myelin debris; decrease in MCT1 and MBP; and increase in cholesterol esters	Kang et al., 2013; Philips et al., 2013; Cui et al., 2014; Ferraiuolo et al., 2016; Chaves-Filho et al., 2019; Bonfanti et al., 2020; Peric et al., 2021; Yusuf et al., 2022
Cultured oligodendrocytes of *SOD1^G93A^* mice	Cells carrying human G93A *SOD1* mutation	Decrease in number and extension of branched processes			Bonfanti et al., 2020
*SOD1^G93A^* rats	Rats carrying human G93A *SOD1* mutation			Desorganisation and decompaction; and decrease in PLP, DM-20, phospholipids and cholesterol but no alteration in MBP	Niebroj-Dobosz et al., 2007
*SOD1^G93A^* zebrafish	Zebrafish carrying human G93A *SOD1* mutation	Expression of apoptotic marker	Aberrant proliferation		Kim et al., 2019
*Fus*^Δ*NLS*^ mice	Mice expressing truncated FUS lacking nuclear localization signal	FUS cytoplasmic delocalisation and increase in number in spinal cord white matter		Decreased g-ratio; and decrease in myocilin, *Pmp2* and *Prx* mRNA	Scekic-Zahirovic et al., 2017
Oligo-*Fus*-KO mice	Mice lacking oligodendrocyte *Fus*			Decreased g-ratio; and increase in cholesterol and HMGCR	Guzman et al., 2020
Oligo-*Tardbp*-KO mice	Mice lacking oligodendrocyte *Tardbp*	Decrease in number in gray matter		Decrease in *Mbp*, *Mag*, *Mog*, and *Cnp* mRNA	Ho et al., 2021
*Tardbp^WT^* mice	Mice expressing wild-type *Tardbp*	Increase in TDP43 and expression of apoptotic marker		Demyelination in spinal cord white matter and decrease in MBP	Yang et al., 2022
*PFN1^C71G^* mice	Mice carrying human C71G profilin mutation	MBP and PLP inclusions			Yusuf et al., 2022
*Tmem106b*-KO mice	Mice lacking *Tmem106b*	Decrease in number		PLP1 delocalization	Zhou et al., 2020

#### 3.1.1 TDP43 aggregates

Cytoplasmic inclusions containing TDP43 are typically observed in 95% of ALS and 50% of FTD patients, regardless of the presence or absence of *TARDBP* mutations (Bright et al., 2021). These aggregates were found in sporadic and familial ALS, with oligodendrocytes frequently appearing as the most affected cells (Philips et al., 2013; Brettschneider et al., 2014; Rohan et al., 2014; Fatima et al., 2015). In particular, oligodendroglial TDP43 inclusions were specifically associated with mutations in several genes linked to ALS (e.g., angiogenin, *C9orf72* and optineurin) or FTD (e.g., *C9orf72*, progranulin and sequestosome 1) (Seilhean et al., 2009; Kovacs et al., 2016). Moreover, the aggregates in the frontal cortex were more abundant in ALS patients carrying a *C9orf72* repeat expansion mutation compared to sporadic cases (Lorente Pons et al., 2020). An increased oligodendroglial aggregation of TDP43 also correlated with a more severe form of FTD (Ho et al., 2021). In accordance with these findings, mice overexpressing wild-type *Tardbp* (*Tardbp^WT^* mice) exhibited an increase in TDP43 content in the spinal cord that was higher in glial cells than in neurons, highlighting the importance of the TDP43 proteinopathy for astrocytes and oligodendrocytes (Yang et al., 2022).

Several studies conducted on ALS patients showed that gray matter oligodendrocytes had more TDP43 inclusions than white matter oligodendrocytes suggesting that the aggregates could propagate from gray to white matter cells. In addition, the inclusions were observed in myelinating oligodendrocytes but not in immature or perineuronal non-myelinating oligodendrocytes, suggesting that these cells, being resistant to protein aggregation, could be affected by the disease in a different manner (Brettschneider et al., 2014; Fatima et al., 2015). Another study, however, did not find any difference between white and gray matter TDP43 pathology in the corticospinal tracts of ALS patients (Rohan et al., 2014). Further to this, an ALS case carrying an optineurin mutation showed hyperphosphorylated TDP43 predominantly accumulated in white matter oligodendrocytes (Nolan et al., 2021). The recruitment of patients at different disease stages could explain these *a priori* contradictory findings.

#### 3.1.2 FUS aggregates

Neuronal cytoplasmic inclusions containing FUS are generally associated with early onset and fast progression of ALS and FTD. Unsurprisingly FUS aggregates were also observed in the cytoplasm of oligodendrocytes in ALS patients carrying a *FUS* mutation. The morphology of these inclusions appeared heterogeneous (including round, crescentic or flame-shaped), and occasionally extending into single or ramified processes. Consistent with these findings, FUS accumulated in the cytoplasm of oligodendrocytes of mice expressing a truncated form of FUS lacking the nuclear localization signal (*Fus*^Δ*NLS*^ mice) (Scekic-Zahirovic et al., 2017). Similarly, 10% of FTD patients showed oval or flame-shaped cytoplasmic glial inclusions, which were tested positive for FUS and ubiquitin. Interestingly, these cases did not carry *FUS* mutations, and although the hippocampus was damaged, the frontal and temporal lobes were affected only moderately (Neumann et al., 2009).

#### 3.1.3 *C9orf72* RNA foci

Amyotrophic lateral sclerosis (ALS) and FTD patients carrying a *C9orf72* repeat expansion mutation typically show neuronal accumulations of the expanded RNA repeats into cytoplasmic RNA foci. These aggregates were also observed in oligodendrocytes. Indeed, the burden of cytoplasmic RNA foci per cell was heavier in neurons and oligodendrocytes than in other cells (DeJesus-Hernandez et al., 2011; Mizielinska et al., 2013). However, although aggregates containing dipeptide repeat proteins generated by repeat-associated non-ATG (RAN) translation are typically found in neurons (Ash et al., 2013), it is currently not clear if this kind of inclusions are also present in oligodendrocytes.

#### 3.1.4 SOD1 aggregates

Inclusions of misfolded SOD1 were found in IPSC-derived oligodendrocytes obtained from sporadic and familial cases of ALS carrying a *SOD1* mutation but they were absent from oligodendrocytes of those carrying a *C9orf72* repeat expansion (Ferraiuolo et al., 2016). These inclusions consisted of poorly oriented filaments containing misfolded SOD1 and were observed in the cytoplasm, particularly in the perikaryon, as well as extracellularly, in the periaxonal space between the myelin sheath and the axon (Stieber et al., 2000). In contrast to TDP43, SOD1 aggregates were less abundant in oligodendrocytes than in other cells (Forsberg et al., 2011). Misfolded SOD1 aggregates were also found in oligodendrocytes of mice overexpressing the ALS-linked *SOD1* mutation G93A (*SOD1^G93A^* mice) (Ferraiuolo et al., 2016). Of note, these mice, as well as mice carrying the ALS-linked profilin mutation, also displayed aggregates containing MBP and PLP, two major proteins of the myelin sheath. These inclusions were localized in the spinal cord and increased in size and number as the disease progressed (Yusuf et al., 2022). At present, it is not known whether these myelin proteins also appear in the pathological inclusions of ALS and FTD patients.

#### 3.1.5 Tau protein aggregates

Inclusions containing Tau protein were observed in oligodendrocytes of FTD patients, independently of the presence or absence of *MAPT* mutations. These aggregates consisted of coiled structures of filamentous and tubular material (Kahlson and Colodner, 2016; Hosokawa et al., 2017). Strikingly, the accumulation of Tau protein in oligodendrocytes appeared specific to FTD, since its accumulation preferentially in neurons was rather associated with Alzheimer’s disease (Richter-Landsberg and Bauer, 2004).

### 3.2 Degeneration of mature oligodendrocytes

Whether oligodendrocytes degenerate in ALS and FTD is a matter of controversy. IPSC-derived oligodendrocytes obtained from sporadic and familial ALS patients did not display any sign of degeneration, suggesting that their death, if it occurs, may depend on the disease cellular environment (Ferraiuolo et al., 2016). In fact, gray matter oligodendrocytes in the ventral spinal cord of *SOD1^G93A^* mice and zebrafish did express markers of apoptosis (Philips et al., 2013; Kim et al., 2019). In addition, the death of these cells started before the onset of overt disease, with only less than half of the oligodendrocytes produced during the first post-natal months surviving by the end stage, causing a decrease in the absolute number of mature oligodendrocytes in both gray and white matter (Kang et al., 2013; Bonfanti et al., 2020). Similarly, the specific deletion of *Tardbp* in mouse oligodendrocytes (oligo-*Tardbp*-KO mice) reduced the number of gray matter oligodendrocytes that reach the symptomatic stage, although it did not affect white matter oligodendrocytes (Ho et al., 2021). Oligodendroglial degeneration also occurred in *Tardbp^WT^* mice, as deduced from the localization of the pro-apoptotic factor caspase-3 in cells positive for the oligodendrocyte marker adenomatous polyposis coli (Yang et al., 2022).

It has also been postulated that the proliferation and differentiation of OPCs could compensate for the loss of mature oligodendrocytes (Philips et al., 2013; Cui et al., 2014). In support of this notion, loss of cells positive for oligodendrocyte transcription factor 2 (OLIG2) that was observed in the corpus callosum of a FTD mouse model lacking the gene encoding the lysosomal transmembrane protein 106B (TMEM106B), affected differentiated but not undifferentiated oligodendrocytes. It was proposed that these undifferentiated cells represented a population of reactive precursors trying to replace the lost oligodendrocytes (Zhou et al., 2020). Perhaps more surprisingly, the number of mature oligodendrocytes was unaltered when knocking out *Fus* specifically in mouse oligodendrocytes (oligo-*Fus*-KO mice) (Guzman et al., 2020), and *Fus*^Δ*NLS*^ mice even showed an increase in the number of white matter oligodendrocytes in the ventral horns of the spinal cord (Scekic-Zahirovic et al., 2017). Therefore, based on the amounts of mature oligodendrocytes, it appears that the population of oligodendroglial cells is affected by the lack of FUS and/or delocalization of its truncated form in a manner different from that observed in models having other disease-linked gene alterations.

## 4 Pathological hallmarks of OPCs in ALS and FTD

As mentioned above, several studies postulated an increase in the proliferation of OPCs as a means of compensating mature oligodendroglial degeneration. Thus, OPCs positive for the oligodendrocyte precursor cell marker NG2 appeared activated in the motor cortex and spinal cord of ALS patients (Kang et al., 2013). In addition, the transcriptomic analysis of samples from frontal and temporal cortex of FTD patients with TDP43 proteinopathy revealed a gene expression signature indicative of increased oligodendroglial activity (Hasan et al., 2022). These results were corroborated in *SOD1^G93A^* mice and zebrafish, where an excess of OPC proliferation was observed in the gray matter of the ventral spinal cord at a pre-symptomatic age, as well as later, though less importantly, in the white matter (Guan et al., 2007; Kang et al., 2013; Philips et al., 2013; Kim et al., 2019; Bonfanti et al., 2020). However, in oligo-*Tardbp*-KO mice, OPCs were shown to proliferate preferentially in the white matter of the spinal cord at pre-symptomatic and end stages (Wang et al., 2018). These differences in preferential proliferation between white and gray matter could be due to the fact that the regeneration capacity of gray matter oligodendrocytes tends to decline in an age-dependent manner (Ho et al., 2021), likely making OPC proliferation in the gray matter only present in younger animals. In contrast with these findings, OPCs appeared unaltered in oligo-*Fus*-KO mice (Guzman et al., 2020). Moreover, the increase in the number of mature oligodendrocytes observed in the white matter of end stage *Fus*^Δ*NLS*^ mice occurred without any mature oligodendrocyte degeneration, which suggests that the presumed proliferation of OPCs preceding the increase in mature oligodendrocytes could serve purposes other than compensating oligodendrocyte cell death (Scekic-Zahirovic et al., 2017).

Together with an abnormal rate of proliferation, the differentiation of OPCs into mature cells could also be affected in ALS and FTD. Newly born OPCs in the spinal cord of *SOD1^G93A^* mice failed to differentiate into fully mature myelinating oligodendrocytes, resulting in immature and dystrophic cells unable to provide efficient myelination or metabolic support to axons (Kang et al., 2013; Bonfanti et al., 2020). With thicker and more elongated cell bodies, these immature oligodendrocytes were detectable before neuronal loss and tended to increase in number upon disease progression, with almost all being completely dysmorphic by the end stage (Philips et al., 2013; Cui et al., 2014; Peric et al., 2021). Consistent with these morphological alterations observed *in vivo*, cultured oligodendrocytes from *SOD1^G93A^* mice also displayed a reduction in the number and extent of branched processes (Bonfanti et al., 2020).

## 5 Altered myelin in ALS and FTD

**FIGURE 1:**
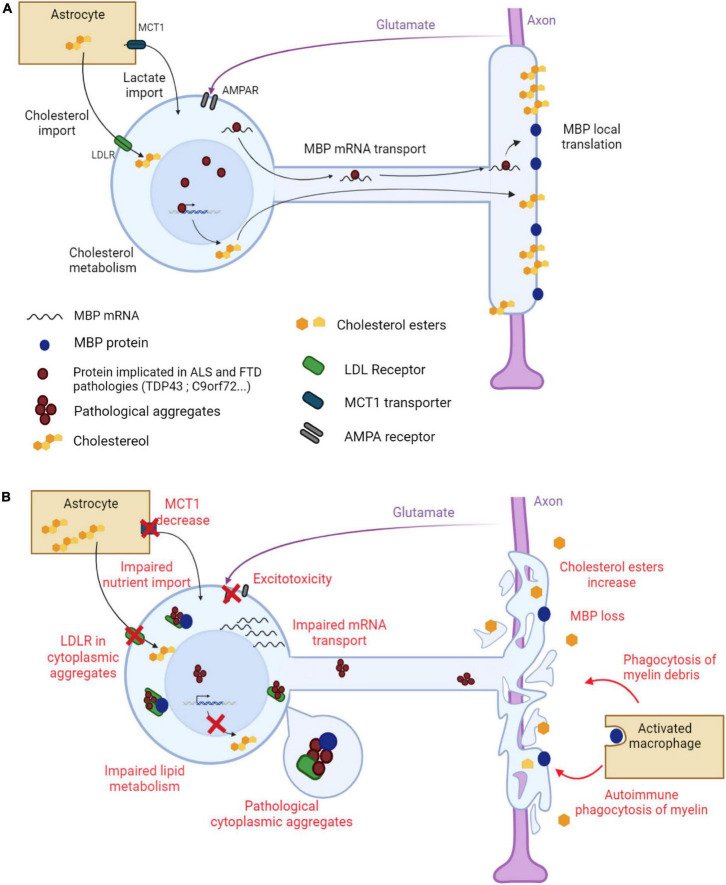
Main mechanisms underlying myelin alterations in ALS and FTD. **(A)** Myelination in a healthy oligodendrocyte. The myelin sheath is made of proteins, such as MBP, and lipids, cholesterol being the most abundant. *MBP* mRNA is transported along oligodendroglial protrusions toward the myelin sheath where it is locally translated. Cholesterol is synthesized by the oligodendrocyte but is also provided by neighboring astrocytes through oligodendroglial LDL receptors. Astrocytes also provide metabolites to the oligodendrocyte through the monocarboxylate transporter MCT1. The oligodendrocyte receives glutamate signals from the axon through AMPA receptors to adapt the ensheathment to axonal needs. **(B)** Altered myelination in an ALS/FTD oligodendrocyte. Pathological aggregates form in the cytoplasm of the oligodendrocyte. LDL receptors, together with other proteins, are trapped in these aggregates, leading to impaired cholesterol import. Oligodendroglial lipid metabolism is also impaired, leading to a global decrease in lipid content in the myelin sheath. *MBP* mRNA transport toward the myelin sheath is altered resulting in a decrease in MBP content. MBP is also trapped in cytoplasmic aggregates. The myelin sheath degenerates and myelin debris stimulate phagocytosis. Decreased MCT1 expression results in impaired nutrient import into the oligodendrocyte. Oligodendroglial AMPA receptor dysregulation triggers aberrant Ca^2+^ permeability and glutamate excitotoxicity. Created with BioRender.com.

Although ALS and FTD are not considered as pure demyelinating diseases, increasing evidence strongly suggests that the myelin sheath shows important modifications in terms of (ultra)structure and chemical composition. These modifications have been studied in patients and animal models (Tables 1, 2 and Figure 1).

### 5.1 Alterations in myelin structure

Diffuse myelin pallor, spongiosis and demyelination were observed in the spinal cord, brainstem, motor cortex and subcortical white matter of patients with sporadic and familial ALS (Hayashi et al., 2001; Kang et al., 2013; Nolan et al., 2021). Of note, the gray matter was more affected in the motor cortex than in the ventral spinal cord (Kang et al., 2013). In contrast, these findings were not confirmed in *Tardbp^WT^* mice since demyelination rather appeared in the white matter, mainly in the lumbar spinal cord, and became less severe toward the rostral areas of the brain (Yang et al., 2022). These observations emphasize, once again, the differences between white and gray matter as well as between cortical and spinal cord involvement. It should be mentioned, however, that other studies did not corroborate these findings. Spectral confocal reflectance imaging of samples from sporadic and familial ALS cases showed a normal compact myelin and no changes in myelin density in at least the motor cortex white matter (Sadler et al., 2022).

The main myelin alteration in the motor cortex of ALS patients was the presence of water, both inside and outside the myelin sheath (Kolind et al., 2013). A similar alteration was observed in the spinal cord of *SOD1^G93A^* mice and rats even at a pre-symptomatic stage, accompanied by the vacuolization and detachment of myelin lamellae eventually leading to the loss of compaction and destruction of the myelin sheath, and to the accumulation of myelin debris around degenerating axons suggestive of Wallerian degeneration. Moreover, the severity of these modifications increased as the disease progressed (Niebroj-Dobosz et al., 2007; Kang et al., 2013). The myelin sheath in the gray matter of the ventral spinal cord of *SOD1^G93A^* and *Fus*^Δ*NLS*^ mice also exhibited a lower ratio of the inner-to-outer diameter of myelinated axons (also referred to as the g-ratio), suggesting an abnormally thick myelin sheath and potential disturbances to axon function (Kang et al., 2013; Scekic-Zahirovic et al., 2017).

### 5.2 Alterations in myelin lipid composition

High levels of cholesterol esters were detected in the white matter of the cortex and spinal cord of sporadic ALS patients, with this increase also correlating to a shorter disease duration (Cutler et al., 2002; Sadler et al., 2022). This alteration was also observed in the spinal cord of *SOD1^G93A^* mice (Chaves-Filho et al., 2019). Similarly, an accumulation of cholesterol esters was found in the frontal and parietal white matter of FTD patients carrying a progranulin mutation, although it was not observed in *C9orf72* repeat expansion carriers. Since excess cholesterol typically accumulates in the form of esters, the increased amounts of these lipids could result from myelin breakdown (Marian et al., 2023). In contrast to these findings, oligo-*Fus*-KO mice showed an increase in cholesterol in the corpus callosum, associated with an increase in the expression of 3-hydroxy-3-methylglutaryl-CoA reductase (HMGCR), the rate-limiting enzyme for cholesterol synthesis, which suggests the formation rather than destruction of myelin. In accordance with this hypothesis, these mice exhibited a decreased g-ratio, indicating thicker myelin sheaths (Guzman et al., 2020).

Low levels of sphingomyelin and ceramides were found in the cortical white matter of patients with sporadic and familial ALS (Sadler et al., 2022). Conversely, significant amounts of these lipids were detected in the cerebrospinal fluid, suggesting a progressive destruction of the myelin sheath (Blasco et al., 2017). A decreased content of sphingolipids was also observed in the hippocampus of FTD patients carrying the risk allele of *TMEM106B* (Lee et al., 2023), as well as in the frontal cortex white matter of FTD patients carrying the progranulin or *C9orf72* repeat expansion mutation (Marian et al., 2023). In contrast, levels of sphingomyelin were increased in the spinal cord white matter of ALS patients, and in pre-symptomatic and symptomatic *SOD1^G93A^* mice (Cutler et al., 2002), highlighting clear-cut differences in terms of lipid alterations in different areas of the CNS.

### 5.3 Alterations in myelin protein composition

The studies investigating the protein composition of myelin in ALS and FTD reported contrasting changes at the mRNA and protein level. An integrative transcriptomic analysis of *post-mortem* spinal cord samples from ALS and ALS/FTD patients revealed a down-regulation of the expression of several genes that were specifically ascribed to the oligodendrocyte cell type; in particular, a decrease in the expression of *MOBP*, involved in myelin compaction, was observed (Humphrey et al., 2023). The expression of *Mbp*, *Mag*, *Mog*, and *Cnp* was also decreased in end stage oligo-*Tardbp*-KO mice, along with *HMGCR* in patients with TDP43 proteinopathy (Ho et al., 2021). Additional genes, characteristic of peripheral myelin, such as myocilin, *Pmp2* and *Prx*, were also down-regulated in the spinal cord of *Fus*^Δ*NLS*^ mice (Scekic-Zahirovic et al., 2017). On the other hand, symptomatic oligo-*Fus*-KO mice showed an increase in the expression of *Hmgcr* but no changes in other major myelin genes were detected (Guzman et al., 2020).

The alterations in gene expression mentioned above did not always correlate with changes in protein content, suggesting different mechanisms of dysregulation at transcriptional, translational and/or post-translational level. MBP content was reduced in the gray matter of the motor cortex and spinal cord of patients with sporadic and familial ALS, specifically carrying a *SOD1* or *C9orf72* repeat expansion mutation (Kang et al., 2013; Rohan et al., 2014). This was not observed in the cortical white matter of sporadic or familial ALS cases (Sadler et al., 2022), consistent with the general trend that gray matter seems to be more affected than white matter. Decreased MBP levels were also found in the white and gray matter of the frontal cortex of FTD patients carrying a progranulin or *C9orf72* repeat expansion mutation but not in sporadic cases (Lorente Pons et al., 2020; Sirisi et al., 2022; Marian et al., 2023). The decrease in MBP was associated with a reduction in the contents of other major myelin proteins, including CNP and PLP, in FTD patients carrying a progranulin (but not *C9orf72* repeat expansion) mutation, as well as in ALS patients carrying a *TARDBP* mutation (Kang et al., 2013; Marian et al., 2023). Finally, MBP levels were decreased in *Tardbp^WT^* mice (Yang et al., 2022) but unaffected in *SOD1^G93A^* rats (Niebroj-Dobosz et al., 2007).

## 6 Mechanisms of oligodendroglial pathology

### 6.1 Cell-autonomous alterations in myelin and oligodendrocytes

Several studies have interrogated whether oligodendrocytes are affected by ALS and FTD in a cell-autonomous manner. Abnormal aggregates are well known to perturb numerous cellular functions, and this can be especially relevant for oligodendrocytes in which the accumulation of certain pathogenic factors is particularly enhanced, as compared to other cell types. Thus, the expression of *C9orf72*, which is normally high in oligodendrocytes, was increased specifically in regions affected by ALS, hence favoring the toxic accumulation of expanded *C9orf72* RNA repeats particularly in oligodendrocytes (Langseth et al., 2017). In contrast, mature oligodendrocytes contained less misfolded SOD1 inclusions than other cell types (Forsberg et al., 2011), possibly due to the fact that oligodendrocytes highly express the heat shock protein αB-crystallin, which prevents the conversion of soluble SOD1 into insoluble forms prone to aggregate (Wang et al., 2005). Despite such a protective mechanism, SOD1 still accumulated in oligodendrocytes of *SOD1^G93A^* zebrafish associated with fluid vacuoles located between decompacted myelin lamellae (Kim et al., 2019). These vacuoles also appeared in oligo-*Tardbp*-KO mice, combined with a decrease in myelin sheath thickness and the down-regulated expression of several major myelin genes, including *Mbp*, *Plp*, *Mog*, and *Mag*, which resulted in a concomitant reduction in the content of at least MBP and MOG. Interestingly, these alterations occurred in the gray and white matter of the spinal cord even in the absence of motor neuron cell death and were sufficient to trigger a progressive decrease in muscle strength and motor coordination together with a shortened lifespan (Wang et al., 2018). Another mouse model of ALS obtained by knocking out optineurin in mature oligodendrocytes also exhibited abnormal myelination in the ventrolateral white matter of the spinal cord (Ito et al., 2016). Likewise, oligo-*Fus*-KO and *Fus*^Δ*NLS*^ mice showed FTD-like behavioral alterations, mainly hyperactivity and disinhibition, which correlated with an increase in myelin thickness without signs of neuronal death (Scekic-Zahirovic et al., 2017, 2021; Guzman et al., 2020). Taken together, these findings point to cell-autonomous alterations occurring specifically in oligodendrocytes that very likely contribute to the whole disease picture.

### 6.2 Mechanisms triggering mature oligodendrocyte cell death

Several mechanisms have been postulated to explain the degeneration of mature oligodendrocytes in ALS and FTD (Ito et al., 2009; Iuchi et al., 2021). Firstly, oligodendrocytes can die due to the loss of function of key proteins necessary for their survival that frequently appear trapped in the cytoplasmic inclusions characteristically seen in these conditions. Deleting *Tardbp* expression in cultured oligodendrocytes triggered cell death through the activation of a necroptosis mechanism mediated by receptor-interacting protein kinase 1, which highlights the essential role of TDP43 in oligodendrocyte survival (Wang et al., 2018; Ho et al., 2021). Strikingly, deleting *Tardbp* expression *in vivo* specifically in astrocytes also triggered a selective reduction in the number of mature oligodendrocytes, and it was suggested that the loss of TDP43 could contribute to kill oligodendrocytes (and neurons) by modifying the gene expression program of astrocytes toward a pro-inflammatory phenotype (Peng et al., 2020).

Mature oligodendrocytes are sensitive to glutamate excitotoxicity and, like neurons, they can die from excessive glutamate exposure (Xu et al., 2008). Normally, OPCs express Ca^2+^-permeable AMPA receptors that become impermeable upon differentiation to mature oligodendrocytes. This transition was impaired in oligodendrocytes from ALS patients carrying a *Tardbp* mutation, rendering AMPA receptors still permeable to Ca^2+^ and enhancing vulnerability to excitotoxicity (Barton et al., 2021). Under physiological conditions, free fatty acids, such as oleic acid and linoleic acid, help to inhibit glutamate-induced cell death. The levels of oleic acid and linoleic acid stayed low in the plasma of *SOD1^G93A^* mice prior to disease onset, which could be related to a higher vulnerability to excitotoxicity. In fact, feeding *SOD1^G93A^* mice with a cocktail rich in oleic acid and linoleic acid suppressed oligodendrocyte cell death in the spinal cord of these mice, further reinforcing the link between glutamate excitotoxicity and mature oligodendrocyte degeneration (Maruyama et al., 2023).

Finally, not all the alterations affecting proteins essential for maintaining the activity of oligodendrocytes triggered their degeneration. The decreased expression of the monocarboxylate transporter MCT1 observed in sporadic ALS patients and *SOD1^G93A^* mice was implicated in affecting the metabolic support of axons by oligodendrocytes, causing axonal damage and neuronal loss. However, such a decrease in MCT1 expression did not cause any death of cultured oligodendrocytes, and oligodendrocytes from heterozygous MCT1 null mice did not show any change in morphology and number (Lee et al., 2012).

### 6.3 Mechanisms triggering impaired OPC proliferation and differentiation

Several mechanisms have been proposed to contribute to the impairment of OPC differentiation seen in ALS and FTD. Under physiological conditions, the expression of G protein-coupled receptor 17 (*GPR17*) is normally down-regulated to enable the differentiation of OPCs. Increasing the expression of this receptor is enough to block the maturation process (Fumagalli et al., 2015). GPR17 levels appeared increased in the spinal cord of *SOD1^G93A^* mice even at a pre-symptomatic stage. In addition, the percentage of mature cells derived from cultured OPCs obtained from *SOD1^G93A^* mice was increased by blocking GPR17. Overall, these findings suggest a role for GPR17 in ALS (Bonfanti et al., 2020). Erb-B2 receptor tyrosine kinase 4 (*ERBB4*) is known to play an important role in oligodendrocyte maturation and is also involved in myelin formation. Interestingly, several mutations in *ERBB4* were identified as causing late-onset ALS, and the expression of *ERBB4* was decreased in the spinal cord of sporadic cases (Takahashi et al., 2019). Since *ERBB4* expression is in part controlled by interacting with TDP43, it was postulated that the cytoplasmic delocalization of TDP43, typically observed in ALS and FTD, could affect *ERBB4* mRNA transport and/or translation and hence impair oligodendrocyte maturation (Schwenk et al., 2016). Von Hippel Lindau protein (VHL) is another key player in OPC differentiation and myelination (Yuen et al., 2014), shown to colocalize with phosphorylated TDP43 in cytoplasmic inclusions of OPCs and oligodendrocytes in the spinal cord of sporadic ALS patients. This interaction was more defined in these cells than in any other cell type and was promoted in the presence of misfolded TDP43. In fact, VHL and TDP43 stabilized and enhanced the formation of perinuclear inclusions of each other, thus preventing their degradation. A similar mechanism was found to occur with mutant SOD1. It was proposed that the aggregates of phosphorylated TDP43 stimulate the accumulation of VHL in insoluble fractions, and hence limits OPC differentiation and myelination (Uchida et al., 2016). Finally, notch receptor 1 (NOTCH1) is an important negative regulator of OPC differentiation, and its expression was decreased in ALS patients and *SOD1^G93A^* mice (Zhang et al., 2009). However, follow-up studies did not corroborate these findings (Liu et al., 2020), and the conditional deletion of *Notch1* in OPCs failed to improve oligodendroglial function and disease outcome in *SOD1^G93A^* mice (Eykens et al., 2018).

Oxidative stress has been shown to play a crucial role in ALS and FTD (Motataianu et al., 2022). Regarding oligodendrocytes, oxidative stress impaired the maturation of OPCs not only by decreasing the expression of genes promoting their differentiation, such as SRY-box transcription factor 10 and *OLIG2*, but also by increasing the expression of genes known to inhibit it, such as the family of inhibitors of DNA binding and cell differentiation (Id) genes. Oxidative stress also affected the deacetylation of histones necessary to initiate OPC differentiation (French et al., 2009). Lastly, since the process of differentiation into mature oligodendrocytes can be interrupted through the activation of a toll-like receptor 3-dependent pathway, it was postulated that the implication of pro-inflammatory mechanisms, commonly observed in ALS and FTD, could alter, at least in part, OPC differentiation (Boccazzi et al., 2021).

### 6.4 Mechanisms affecting the myelination process

Besides the loss of mature oligodendrocytes and the impairment in OPC maturation, the formation and maintenance of the myelin sheath can also be affected in ALS and FTD (Figure 1). In agreement with this notion, the reduction in the content of MBP in the spinal cord of ALS and FTD patients with TDP43 proteinopathy did not correlate with the decrease in the number of mature oligodendrocytes, which strongly suggests that certain myelin modifications can take place, at least in part, as an independent event (Rohan et al., 2014). The fact that myelin defects were observed around uninjured axons in *SOD1^G93A^* mice also suggests that the alterations of the myelin sheath may occur prior to motor neuron loss (Kang et al., 2013). Several mechanisms have been proposed to explain the modifications of the myelin sheath observed in ALS and FTD.

Several genes whose mutations have been linked to ALS and/or FTD, such as *TARDBP*, *FUS*, angiogenin and *HNRNP1*, are normally implicated in different aspects of the metabolism of RNA. For example, TDP43 is known to bind to mRNAs encoding major myelin proteins, including MBP, PLP, MOG and MAG. Not surprisingly, these mRNAs were progressively down-regulated in the spinal cord of oligo-*Tardbp*-KO mice (Wang et al., 2018). Similarly, FUS was shown to bind to *Mbp* mRNA, and knocking out *Fus* produced aberrant splice variants of *Mobp* and *Mag* RNA (Hoell et al., 2011; Lagier-Tourenne et al., 2012). Overall, these findings point to an equilibrium between myelin RNAs and associated RNA binding proteins necessary to ensure proper myelination. Particularly for *MBP* mRNA, binding to HNRNPA2 is required for transport to the myelin sheath, where it is then translated. Interestingly, mutant forms of ALS and FTD-linked TDP43 and *C9orf72* were shown to trap HNRNPA2, likely compromising the transport of *Mbp* mRNA toward the myelin sheath (Buratti et al., 2005; DeJesus-Hernandez et al., 2011; Lorente Pons et al., 2020). Consistent with these findings, the trafficking of *MBP* mRNA was altered in ALS patients carrying a *C9orf72* repeat expansion mutation, although this alteration did not cause any noticeable modification in the general structure of myelin (Barton et al., 2021). In oligodendrocytes, as in neurons, microtubule dynamics and stabilization are essential not only to allow the formation of cellular processes and myelin extensions but also to assure mRNA transport. The microtubule-associated protein Tau, which appears frequently aggregated in cases of FTD, is involved in these processes. Thus, the oligodendroglial knock-down of *Mapt*, the gene encoding Tau protein, triggered the retention of *Mbp* mRNA in the cell soma, causing a decrease in peripheral *Mbp* expression and subsequent impaired myelination (Carson et al., 1997; Seiberlich et al., 2015). Finally, the analysis of the brain transcriptome in a mouse model of FTD lacking *TMEM106B* showed a generalized down-regulation of genes implicated in myelination and axonal ensheathment. This was associated with the delocalization of the myelin protein PLP1 in lysosomes instead of being properly assembled into the membrane sheath, indicating the importance of TMEM106B for PLP1 transport (Zhou et al., 2020).

Cholesterol is the most present lipid component of the myelin sheath. Under physiological conditions, TDP43 binds to the mRNAs encoding HMGCR and sterol regulatory element binding transcription factor 2 (SREBF2) and promotes the expression of these genes involved in cholesterol synthesis. The expression of *HMGCR* was reduced in oligodendrocytes of FTD patients with TDP43 proteinopathy, and that of *Srebf2* was also decreased in oligo-*Tardbp*-KO mice. However, re-expressing *Srebf2* in these mice restored cholesterol levels and protected against the demyelination caused by the lack of TDP43 (Ho et al., 2021). Many genes implicated in cholesterol metabolism also display binding motifs for FUS but, in contrast to what was observed with TDP43, the expression of *Hmgcr* was rather increased in oligo-*Fus*-KO mice concomitantly with an increase in cholesterol content and myelin thickness (Guzman et al., 2020). Oligodendrocytes need large amounts of cholesterol for the formation and maintenance of the myelin sheath. The import of cholesterol from astrocytes mediated by low-density lipoprotein receptor (LDLR) is one of the mechanisms making cholesterol available to oligodendrocytes. TDP43 co-aggregated with LDLR in oligodendrocytes of FTD patients with TDP43 proteinopathy, and the expression of LDLR itself was decreased in oligo-*Tardbp*-KO mice, suggesting a reduction in the transfer of cholesterol and a defect of myelination. Consistent with these findings, supplementing cultured oligodendrocytes of oligo-*Tardbp*-KO mice with cholesterol restored their myelinating capacity lost in the absence of TDP43 (Ho et al., 2021). Astrocytes also provide oligodendrocytes with lactate, via MCT1, and lactate is then used to synthesize different classes of myelin lipids (Rinholm et al., 2011). The generalized reduction in MCT1 levels observed in the CNS of ALS patients and related animal models could therefore result in deficient intake of lactate by oligodendrocytes and subsequent impaired myelination.

Sphingolipids are also important constituents of the myelin sheath. The presence of the disease risk allele of *TMEM106B* was associated with reduced levels of myelin sphingolipids in the hippocampus of patients with FTD (Lee et al., 2023). Similarly, a loss of myelin sphingolipids was found in FTD patients carrying a progranulin mutation, and a mouse model of FTD lacking progranulin showed changes in the activity of enzymes involved in sphingolipid metabolism, which strongly suggests the implication of such genes in maintaining oligodendroglial function (Huang et al., 2020; Lok and Kwok, 2021).

It has been observed in several neurodegenerative diseases that the structure and function of certain proteins may be affected by post-translational citrullination. Levels of citrullinated proteins, as well as those of the peptidylarginine deiminases responsible for citrullination, were increased in the spinal cord of mice carrying an ALS-linked *SOD1^G93A^* or profilin mutation. Interestingly, these citrullinated proteins formed aggregates containing MBP and PLP. In fact, the citrullination of MBP caused its dissociation from the membrane, and dissociated MBP formed extended structures prone to aggregate or be proteolyzed. It was hypothesized that the proteolysis of such structures could release immunogenic peptides, eventually triggering a kind of autoimmune response and the subsequent destruction of the myelin sheath (Yusuf et al., 2022). Of note, myelin phagocytosis could be also caused by macrophages stimulated in response to abnormally high levels of glycine, as deduced from an increase in glycine content found in the plasma of ALS patients (Carmans et al., 2010).

## 7 Contribution of affected oligodendrocytes to neuronal alterations in ALS and FTD

### 7.1 Impaired axonal function

**FIGURE 2:**
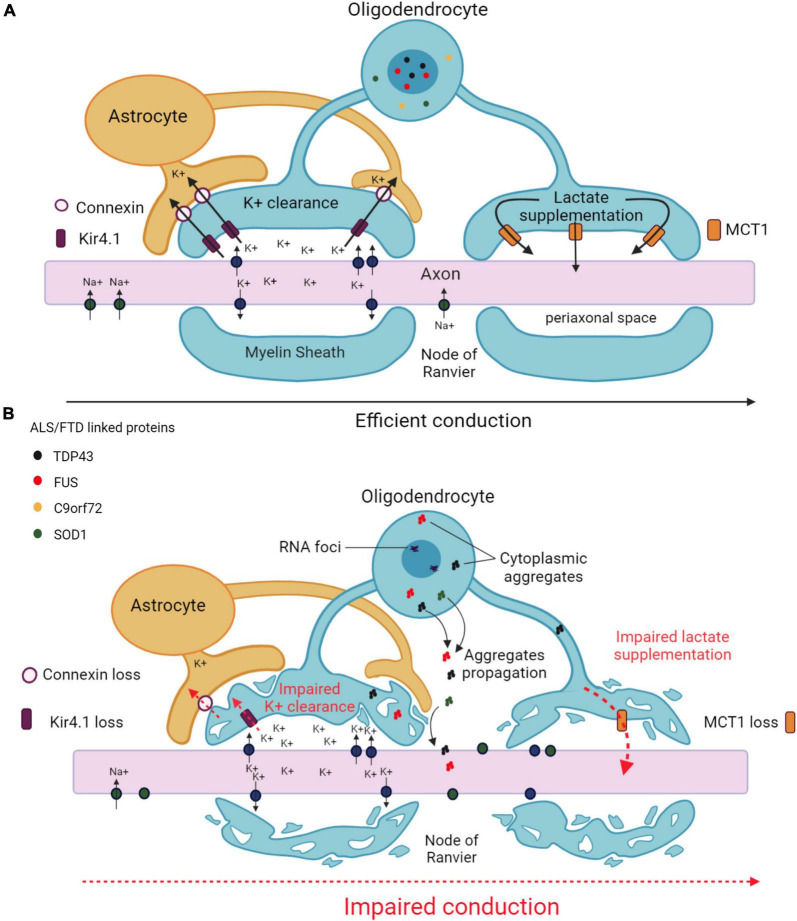
Main mechanisms underlying axonal impairment caused by oligodendrocytes in ALS and FTD. **(A)** Functional interactions between oligodendrocytes and axons in an healthy CNS. Oligodendrocytes ensure K^+^ clearance from the periaxonal space to enable the propagation of action potentials. K^+^ is taken up by the oligodendrocyte through inwardly rectifying Kir4.1 channels, and transferred to the astrocyte through connexins. Oligodendrocytes also provide metabolic support to axons by supplying lactate through MCT1 transporters. **(B)** Altered functional interactions between oligodendrocytes and axons in ALS/FTD. Decreases in Kir4.1 channels and connexin lead to impaired K^+^ clearance in the periaxonal space resulting in impaired action potential generation. Decreased MCT1 expression leads to failure in metabolic supply to the axon. Pathological aggregates formed in the cytoplasm of oligodendrocytes may diffuse from into the axon. All together, these alterations results in axonal damage and degeneration. Created with BioRender.com.

Given the intimate connection with axons, it seems very likely that oligodendrocytes affected by ALS and/or FTD make an impact on axonal structure and function, potentially leading to neuronal cell death (Figure 2). In accordance with this hypothesis, oligodendrocytes derived from ALS patients carrying disease-related mutations in *SOD1*, *TARDBP*, *C9orf72*, or FIG4 phosphoinositide 5-phosphatase induced *in vitro* neuronal hyperexcitability and subsequent motor neuron cell death (Ferraiuolo et al., 2016). Mice and zebrafish expressing *SOD1^G93A^* specifically in mature oligodendrocytes (oligo-*SOD1^G93A^* mice and zebrafish) showed a reduction in axonal conduction velocity and neuromuscular transmission, as well as progressive axonal degeneration (Kang et al., 2013; Kim et al., 2019). Another mouse model lacking optineurin in mature oligodendrocytes did not exhibit any defect in the number and morphology of spinal cord motor neurons but showed swollen motor axons and muscle denervation, reminiscent of the axonal pathology observed in ALS patients at early disease stages (Ito et al., 2016). Similarly, fast and slow axonal transport was reduced in mice expressing mutant Tau protein specifically in oligodendrocytes, triggering an age-dependent motor deficit that preceded neurodegeneration (Higuchi et al., 2005). Additional studies showed that mice expressing the FTD-linked *MAPT* mutation P301 exhibited dysmorphic ad-axonal myelin lamellae and impaired conduction of action potentials, which could account for the loss of performance in the object recognition test used to assess learning and memory in these mice (Jackson et al., 2018).

Inwardly rectifying Kir4.1 channels, normally expressed by oligodendrocytes (and astrocytes), are responsible for the clearance of excess accumulation of K^+^ in the extracellular space and contribute to the regulation of neuronal excitability (Schirmer et al., 2018). The expression of Kir4.1 channels was decreased in the spinal cord of *SOD1^G93A^* rats, particularly in regions exhibiting degenerating motor neurons and dysmorphic oligodendrocytes. It was postulated that the reduction of inward currents observed in cultured oligodendrocytes derived from these animals could compromise proper axonal conduction (Peric et al., 2021). The expression of connexins Cx47 and Cx32, implicated in transferring excess extracellular K^+^ between oligodendrocytes and astrocytes, was also reduced in oligodendrocytes in the ventral horns of the spinal cord of *SOD1^G93A^* mice, especially in those containing accumulated mutant SOD1 (Cui et al., 2014), which further reinforces the notion of a defective buffering of K^+^ in the extracellular environment surrounding motor neurons. Altering the functionality of voltage-gated K^+^ channels represents another way of affecting the propagation of axonal action potentials. Oligo-*SOD1^G93A^* zebrafish developed behavioral and learning abnormalities and motor defects in the early symptomatic stage associated with an impaired axonal conduction capacity, and this phenotype was ameliorated by administering the pan voltage-gated K^+^ channel inhibitor 4-aminopyridine (Kim et al., 2019), highlighting the pathological influence of genetically altered oligodendrocytes on global neuronal activity.

### 7.2 Impaired metabolic support to neurons

Monocarboxylate transporter 1 (MCT1) is the most important monocarboxylate transporter expressed by oligodendrocytes (and astrocytes) that supplies lactate and other metabolites to axons. MCT1 levels were decreased in the CNS of ALS patients and *SOD1^G93A^* mice and zebrafish (Lee et al., 2012; Philips et al., 2013; Ferraiuolo et al., 2016; Kim et al., 2019). Moreover, the expression of *SOD1^G93A^* in human embryonic kidney cells reduced MCT1 content although only at the protein level, indicating the implication of a post-transcriptional mechanism (Philips et al., 2013). Of note, the conditioned medium obtained from cultured oligodendrocytes lacking MCT1 caused motor neuron cell death *in vitro*, which was partly restored upon lactate supplementation (Ferraiuolo et al., 2016). On the other hand, the removal of mutant SOD1 from the oligodendrocyte lineage in *SOD1^G37R^* mice increased MCT1 content, delayed disease onset and prolonged lifespan (Kang et al., 2013). Collectively, these findings suggest that the down-regulation of MCT1 observed in patients and related animal models can affect neuronal function and should not be exclusively considered as a consequence of the loss of oligodendrocytes during the pathological process.

Other studies, however, failed to show any implication of oligodendroglial MCT1 in affecting neuronal function. In fact, cultured oligodendrocytes derived from ALS patients carrying a *Tardbp* mutation did not exhibit any deficit in lactate transport (Barton et al., 2021). In addition, the AAV-mediated delivery of *MCT1* to mature oligodendrocytes did not provide functional rescue nor survival benefit to *SOD1^G93A^* mice (Eykens et al., 2021). Deleting *Mct1* specifically in mouse oligodendrocytes caused a very modest axonal damage not noticed until two years of age (Philips et al., 2021). Finally, an axonopathy similar to that observed in ALS was detected in mice by 8 months of age only after the heterozygous deletion of *Mct1* in all cells (Lee et al., 2012). These findings strongly suggest that the role of MCT1 in supplying metabolic support could result from its expression in other cell types, such as astrocytes. Alternatively, the presence of MCT1 in non-myelinating perineuronal oligodendrocytes, which seem to resist to the pathological process, could also play a relevant role in maintaining the metabolic support to neurons (Rohan et al., 2014).

### 7.3 Neuronal excitotoxicity

Glutamine synthetase is responsible for the synthesis of glutamine, which is in turn converted into glutamate as part of the glutamate/glutamine cycle that maintains appropriate glutamate concentrations to allow neuronal excitability but, at the same time, impede excitotoxicity. Besides the major contribution of astrocytes, it has been recognized that oligodendrocytes can also participate in the regulation of this cycle. Interestingly, the number of mature oligodendrocytes expressing glutamine synthetase was increased in the ventral spinal cord of *SOD1^G93A^* mice from the pre-symptomatic to end stage, and it was suggested that the resulting accumulation of glutamine could lead to the production of glutamate in excess and subsequent excitotoxicity (Haim et al., 2021).

### 7.4 Transfer of pathogenic proteins

The histological analysis of *post-mortem* samples of spinal cord from ALS patients allowed the establishment of four neuropathological stages defined according to the extension of TDP43 inclusions. The regional localization of these inclusions strongly suggested a possible propagation of TDP43 aggregates from gray matter oligodendrocytes to neurons and to white matter oligodendrocytes (Brettschneider et al., 2014; Fatima et al., 2015). Consistent with this notion, the overexpression of *TARDBP* in cultured oligodendrocytes, when the proteasome was inhibited, led to the formation of cytoplasmic aggregates containing TDP43 that were able to spread into contiguous cells (Ishii et al., 2017). *In vivo* studies also reported that the unilateral inoculation of homogenates derived from tauopathy patients in the lateral corpus callosum of mice resulted in the pathological aggregation of Tau protein in oligodendrocytes and the spreading of these aggregates from the injection site to the contralateral corpus callosum (Ferrer et al., 2019). In addition, the time course of the chimeric expression in the mouse spinal cord of fluorescent *SOD1^G85R^* showed the transfer of mutant SOD1 between motor neurons as well as between motor neurons and gray matter oligodendrocytes. Since the highest degree of protein transfer occurred when oligodendrocytes deployed their projections toward axons, it was suggested that oligodendrocytes could mediate the propagation of pathogenic proteins between motor neurons (Thomas et al., 2017). Last but not least, the co-culture of naive motor neurons with oligodendrocytes derived from ALS patients triggered the death of motor neurons likely via cell-to-cell contacts or, at least, when the two cell types were in close vicinity (Ferraiuolo et al., 2016). In fact, mature oligodendrocytes are able to release exosomes containing SOD1 that then are internalized by neurons. Under physiological conditions, this mechanism enhances the neuron’s tolerance toward oxidative stress, but it can be postulated that such a mechanism could also account for cell-to-cell propagation of pathological SOD1 in an ALS context (Fröhlich et al., 2014). Taken together, these findings further reinforce the implication of oligodendrocytes in disease propagation.

## 8 Concluding remarks

We have reviewed our current knowledge of the alterations specific to myelin and oligodendrocytes in ALS and FTD, and several conclusions can be drawn. Oligodendroglial modifications are clearly observed in patients and related animal models, some of them being already detected at pre-symptomatic stages. Gray matter oligodendrocytes appear to be affected earlier than white matter oligodendrocytes. These alterations mainly concern pathological cytoplasmic aggregates containing disease-related proteins, increased expression of apoptotic markers, and changes in the structure and composition of the myelin sheath, notably pertaining to the amounts of MBP and cholesterol. In general, TDP43, SOD1 and other disease-related animal models recapitulate quite well the common features characteristic of a decrease in myelination as seen in patients. However, the FUS-based models rather show increases in myelin thickness and cholesterol, which raises the question as to whether *FUS* mutations orchestrate a different specific disease mechanism while leading to a similar clinical outcome. Finally, the degeneration of mature oligodendrocytes occurs simultaneously with an increase in the proliferation and differentiation of OPCs, as a means to compensate the loss of oligodendrocytes. Unfortunately, this compensatory mechanism does not seem to work efficiently and the newly generated oligodendrocytes become dysfunctional.

Beyond the well-known implication of other types of glial cells, such as astrocytes and microglial cells, the contribution of oligodendrocytes represents a major advance in understanding the pathological process underlying ALS and FTD. Not only do oligodendrocytes suffer from disease by themselves, they also induce morphological and physiological alterations at the axon level, including axonal swelling, perturbed axonal transport and impaired conduction of action potentials, which all together trigger denervation at the neuromuscular junction. Genetic animal models have provided evidence that the alterations in the axon caused by defective oligodendrocytes are sufficient to induce ALS and FTD-like phenotypes, even in the absence of neuronal loss. It is therefore tempting to suggest that the modifications in the structure and composition of the myelin sheath could cause alterations in conduction velocity and signal synchronicity leading to subsequent motor and cognitive deficits prior to overt neuronal degeneration.

The conditions in which the motor and cognitive alterations coexist in the ALS/FTD syndrome are not yet determined. However, the theory of a progressive propagation of the disease from the brain to the spinal cord or vice versa is likely. At least in the case of ALS, it is commonly accepted that neurodegeneration spreads from a focal site to neighboring areas although the precise underlying mechanism remains obscure. Each oligodendrocyte myelinates up to 50 different axons and, for this reason, it can be considered as a link between neurons and a good candidate for spreading the neurodegenerative process. Thus, having a marked oligodendroglial pathology could facilitate the propagation of neurodegeneration between cognitive and motor areas. In accordance with this hypothesis, the presence of oligodendroglial inclusions in FTD or demented patients correlated with the development of motor alterations (Noda et al., 1999; Forno et al., 2002; Arai et al., 2003; Kovacs et al., 2008). Moreover, oligodendrocytes were more affected in ALS patients carrying a *C9orf72* repeat expansion mutation, common to FTD, than patients presenting with only sporadic ALS (Lorente Pons et al., 2020). On the contrary, patients carrying a *SOD1* mutation, which exclusively leads to ALS, showed a less pronounced oligodendroglial pathology as compared to other cell types (Forsberg et al., 2011). Even though assuming oligodendrocytes could act as intermediaries of disease propagation, the precise mechanisms at work in specific CNS regions might not be the same, since it is well-known that there are phenotypic differences in oligodendrocytes in gray versus white matter, as well as in oligodendrocytes in brain versus spinal cord.

Understanding the correlation between the expression of disease genes in the cell types of concern and the clinical outcome represents another area of research. Interestingly, an intense aggregation of TDP43 in the cytoplasm of FTD oligodendrocytes was associated with a more severe form of the disease (Ho et al., 2021). However, the presence of FUS inclusions in the cytoplasm of ALS oligodendrocytes was correlated with later onset and slower progression than when FUS accumulated preferentially in the cytoplasm of neurons (Mackenzie et al., 2011). More surprisingly, although it is known that mutant presenilin typically causes Alzheimer’s disease, the coexistence of such a mutation and nuclear inclusions of ubiquitin in oligodendrocytes triggered FTD (Riudavets et al., 2013). Similarly, while the existence of a tauopathy in neurons usually leads to Alzheimer’s disease, its presence in glial cells caused FTD (Chung et al., 2021). These studies prompt to consider the nature of the oligodendrocyte pathology as part of the criteria to distinguish between subtypes of ALS and FTD, and of neurodegenerative diseases in general.

## Author contributions

MJ: Writing – original draft. LD: Writing – review & editing. J-LG: Supervision, Writing – review & editing.
